# AEGS: identifying aberrantly expressed gene sets for differential variability analysis

**DOI:** 10.1093/bioinformatics/btx646

**Published:** 2017-10-11

**Authors:** Jinting Guan, Moliang Chen, Congting Ye, James J Cai, Guoli Ji

**Affiliations:** 1 Department of Automation, Xiamen University, Xiamen, China; 2 Xiamen Research Institute of National Center of Healthcare Big Data, Xiamen, China; 3 College of Environment and Ecology, Xiamen University, Xiamen, China; 4 Department of Veterinary Integrative Biosciences, Texas A&M University, College Station, TX, USA; 5 Interdisciplinary Program in Genetics, Texas A&M University, College Station, TX, USA; 6 Innovation Center for Cell Signaling Network, Xiamen University, Xiamen, China

## Abstract

**Motivation:**

In gene expression studies, differential expression (DE) analysis has been widely used to identify genes with shifted expression mean between groups. Recently, differential variability (DV) analysis has been increasingly applied as analyzing changed expression variability (e.g. the changes in expression variance) between groups may reveal underlying genetic heterogeneity and undetected interactions, which has great implications in many fields of biology. An easy-to-use tool for DV analysis is needed.

**Results:**

We develop AEGS for DV analysis, to identify **a**berrantly **e**xpressed **g**ene **s**ets in diseased cases but not in controls. AEGS can rank individual genes in an aberrantly expressed gene set by each gene’s relative contribution to the total degree of aberrant expression, prioritizing top genes. AEGS can be used for discovering gene sets with disease-specific expression variability changes.

**Availability and implementation:**

AEGS web server is accessible at http://bmi.xmu.edu.cn:8003/AEGS, where a stand-alone AEGS application can also be downloaded.

## 1 Introduction

Dysregulation of gene expression in relevant tissues or cells is often found to be associated with human diseases. Thus, gene expression analysis has been commonly used to assess dysregulated gene expression and help identify genetic variants conferring the regulatory impact. To this end, differential expression (DE) analysis is widely adapted to characterize gene expression difference between healthy individuals and individuals affected with a disease. Although DE analysis focuses on detecting the difference in gene expression mean between diseased cases and controls, several methods that focus on differential variability (DV) have been developed to characterize within-group expression heterogeneity. DV analysis can be designed for a case–control setting to detect genes differentially variably expressed between different groups. As dysregulated gene expression may be manifested as either a DE or a DV difference between groups, it is, therefore, important to include DV analysis along with DE analysis in disease gene expression studies.

We have proposed a new multivariate analysis method, namely aberrant gene expression analysis, for identifying aberrantly expressed gene sets (Zeng *et al.*, 2015) and have recently extended it to case–control settings, making it a multivariate DV analysis method (Guan *et al.*, 2016). To make this method easy to use, here we develop a tool, named AEGS, to identify **a**berrantly **e**xpressed **g**ene **s**ets associated with a specific disease. We provide two ways of usage: a web service and a stand-alone application. AEGS can be used to identify gene sets more likely to be aberrantly expressed (i.e. more variably expressed) in diseased individuals than in controls. It can also prioritize individual genes of an aberrantly expressed gene set and compare co-expression networks visually between different groups of samples. AEGS is therefore useful in revealing the relationship between gene function and gene expression variability, providing new insights into the genetic and molecular mechanisms of complex disorders. It can be used to characterize the between-individual heterogeneity in gene expression in different tissues or cell types, useful in personalized medicine.

## 2 Methods

**Fig. 1:**
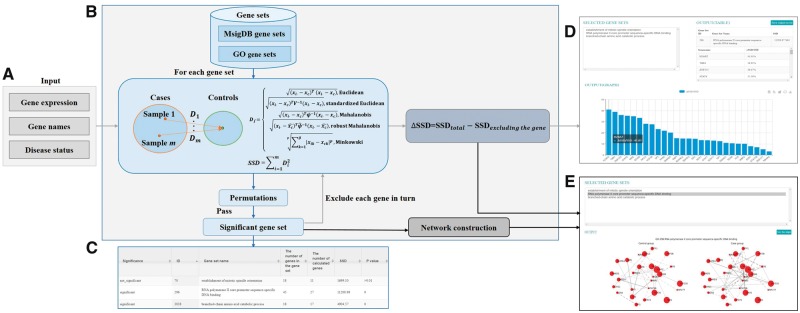
(**A**) The three input files for AEGS. (**B**) The workflow of AEGS including the procedures for identifying significant gene sets, scoring and sorting genes and plotting gene correlation networks. (**C**) The output table of identifying significant gene sets. (**D**) The table and bar char of gene scores. (**E**) Side-by-side comparison of gene co-expression modules using case and control samples

AEGS is developed initially by implementing a multivariate method for identifying aberrantly expressed gene sets, which adopts Mahalanobis distance (MD) (Mahalanobis, 1936) to quantify the dissimilarity in multigene expression vectors between samples. Now AEGS incorporates different kinds of distance metrics, including Euclidean distance, standardized Euclidean distance, MD, robust MD and Minkowski distance. To identify significant gene sets that may be associated with a disease, we first computed the distance from each diseased sample *i* to the multivariate centroid of the controls, denoted as *D_i_* (Fig. 1B). For MD, *D_i_* was computed as: $D_{i}=\sqrt{{\left(x_{i\cdot }-x_{c}\right)}^{T}\psi^{-1}(x_{i\cdot }-x_{c})}$, where *x_i·_* is the gene expression for diseased sample *i*, *x_c_* and *ψ* are expression mean and covariance matrix computed from all controls. For robust MD, only part of controls would be used to compute mean ${\hat{x}}_{c}$ and covariance $\hat{\psi }$ (Fig. 1B). In specific, the algorithm of minimum covariance determinant (Rousseeuw and Van Driessen, 1999) was adopted to subsample *h* observations (*h* = 0.75*n*, where *n* is the number of controls) from which the covariance had the smallest covariance determinant and compute ${\hat{x}}_{c}$ and $\hat{\psi }$ from these *h* controls. For other distance measures, *D_i_* is the distance under corresponding metric from diseased sample *i* to the expression mean of all controls. Then the sum of squared *D_i_* (SSD) was calculated to give a measure of the overall expression variability or dispersion for all cases with respect to controls. To assess the significance of SSD of a given gene set, permutation tests were performed using *N* randomly reconstructed gene sets of the same size. The *P*-value of permutations was determined by *M*/*N*, where *M* is the number of random gene sets having greater SSD than the observed one. Next, to prioritize genes for a given gene set, we calculated ΔSSD to measure the relative contribution of each gene to the overall dispersion. ΔSSD of a gene is the difference between the total SSD value and the one calculated after the gene is excluded (Fig. 1B). Finally, when aberrantly expressed genes are located in modules of the co-expressed network, AEGS can plot the network modules for case and control groups. In such a co-expression network plot, the edge width is proportional to the absolute value of Pearson’s correlation coefficient with a solid line denoting positive correlation and dashed line denoting negative correlation, and the node size is proportional to the ΔSSD value. 

## 3 Results

AEGS offers three main functions: (i) identifying significant gene sets that tend to be aberrantly expressed in a subgroup of samples, (ii) scoring and sorting genes in a gene set by the relative contribution of each gene to the overall dispersion and (iii) plotting co-expression networks for aberrantly expressed gene modules in case and control groups. These functions can be used separately or as an integrated pipeline. The current version of AEGS includes two curated gene sets: molecular signatures database (MsigDB) gene sets (version 5.0) (Liberzon *et al.*, 2011) and gene ontology (GO) term-defined gene sets. Users are required to provide three inputs: a gene expression matrix, a list of gene names and a list of sample disease status (Fig. 1A). After receiving user’s inputs, AEGS starts to scan selected MsigDB or GO terms gene sets to identify significant ones. Users can mark down the job ID for retrieving the result later or use the provided link to check the running progress. A feedback e-mail will also be sent to users once the job is finished. When a user selects several gene sets, AEGS can show whether each set is significant and the result can be downloaded (Fig. 1C). For each gene set from the output list, AEGS can calculate ΔSSD values and then sort genes, as shown in the table and the bar chart (Fig. 1D). AEGS also can be used to visualize and compare gene co-expression networks derived using case and control samples (Fig. 1E). As mentioned, users can use the three main functions separately. For example, after obtaining significant gene sets, users can input a file containing the IDs of these significant gene sets to let AEGS prioritize genes.

## 4 Conclusion

We developed AEGS to identify gene sets aberrantly expressed among a subgroup of samples. It can also be used to prioritize genes and compare co-expression networks. AEGS is a new tool for analyzing the relationship between gene function and gene expression variability.

## Funding

This work was supported by the National Natural Science Foundation of China [61573296, 61473329 and 61673323], the Fujian Provincial Natural Science Foundation of China [2015J01009], the Fundamental Research Funds for the Central Universities in China from Xiamen University (20720170076), and the National Science and Technology Major Project by the Ministry of Industry and Information Technology of China [Application of a new intelligent manufacturing mode of tractors with newly designed wheels 2016-0744].

*Conflict of Interest*: none declared.

## References

[btx646-B1] Guan J. et al (2016) Exploiting aberrant mRNA expression in autism for gene discovery and diagnosis. Hum. Genet., 135, 797–811. 27131873 10.1007/s00439-016-1673-7

[btx646-B2] Liberzon A. et al (2011) Molecular signatures database (MSigDB) 3.0. Bioinformatics, 27, 1739–1740. 21546393 10.1093/bioinformatics/btr260 PMC3106198

[btx646-B3] Mahalanobis P.C. (1936) On the generalised distance in statistics. Proc. Natl. Inst. Sci. India, 2, 49–55.

[btx646-B4] Rousseeuw P.J., Van Driessen K. (1999) A fast algorithm for the minimum covariance determinant estimator. Technometrics, 41, 212–223.

[btx646-B5] Zeng Y. et al (2015) Aberrant gene expression in humans. PLoS Genet., 11, e1004942. 25617623 10.1371/journal.pgen.1004942 PMC4305293

